# Spotlight on the Unusual: A Case Report of Adrenal Angiosarcoma Presenting as an Abdominal Mass

**DOI:** 10.7759/cureus.78968

**Published:** 2025-02-13

**Authors:** Ermilo Echeverría-Ortegón, Javier Casillas, Luis Pech, Isabella Canto

**Affiliations:** 1 School of Medicine, Universidad Marista de Mérida, Mérida, MEX; 2 Radiology, University of Miami Miller School of Medicine, Jackson Memorial Hospital, Miami, USA

**Keywords:** adrenal angiosarcoma, adrenalectomy, adrenal mass, large intra‑abdominal mass, nonspecific abdominal pain

## Abstract

This case discusses the challenges of diagnosing adrenal angiosarcoma, a rare and aggressive malignancy that constitutes an exceptionally small proportion of adrenal tumors. Angiosarcomas originate from blood or lymphatic vessel endothelium, with soft tissue and cutaneous forms being more prevalent than those arising in visceral organs such as the adrenal glands. This report describes a 47-year-old female patient who presented with a large right adrenal mass identified through imaging and was subsequently diagnosed with primary adrenal angiosarcoma. This disease may remain asymptomatic or present with nonspecific symptoms, making early detection challenging. Prompt identification and surgical management are essential, as the tumor exhibits a high propensity for aggressive behavior and metastatic spread. While no standardized treatment guidelines exist, adrenalectomy and systemic therapy are commonly employed strategies. Prognosis remains unfavorable, often influenced by tumor size, necrosis, and metastatic involvement at diagnosis. This case highlights the importance of accurate diagnostic methods and early intervention to enhance patient outcomes.

## Introduction

Angiosarcomas constitute less than 1% of all soft tissue sarcomas; these high-grade malignancies originate from blood and lymphatic vessel endothelium, making them exceedingly rare. Skin and soft tissue angiosarcomas, which commonly occur in the breast, skin, spleen, bone, and liver, are more frequent than those arising in visceral organs or bone, which are often sites of metastasis. Their five-year survival rate typically ranges from 24% to 31% [1-3].

Distinctively, adrenal masses are typically discovered incidentally on computed tomography (CT) of the abdomen, occurring approximately 4% of the time, and in 8% of autopsy series, with benign adenomas accounting for up to 80% of cases [4]. However, primary angiosarcoma of the adrenal gland is an extremely rare malignancy, with fewer than 50 cases reported in the literature. Nonetheless, adrenal angiosarcomas represent a minute fraction of adrenal tumors, and due to the rarity of this disease, their diagnosis can be conventionally challenging for clinicians; if not detected and treated in the early stages, they carry a poor prognosis. The case report aims to highlight the challenges in diagnosing primary adrenal angiosarcomas due to their rarity and emphasize the importance of accurate diagnosis for optimal patient management.

## Case presentation

**Figure 1:**
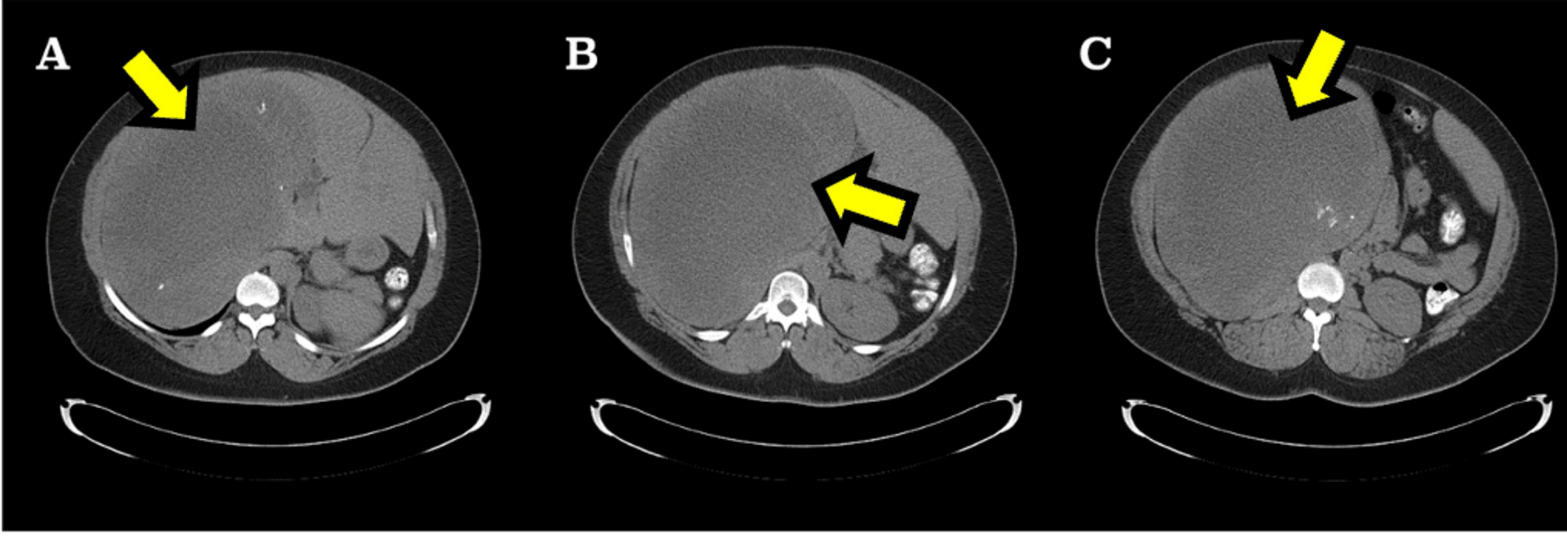
Contrast-enhanced abdominal computed tomography axial images (A-C). A large hypodense mass (arrows) is observed in the right hypochondrium, displacing the liver medially to the left.

**Figure 2:**
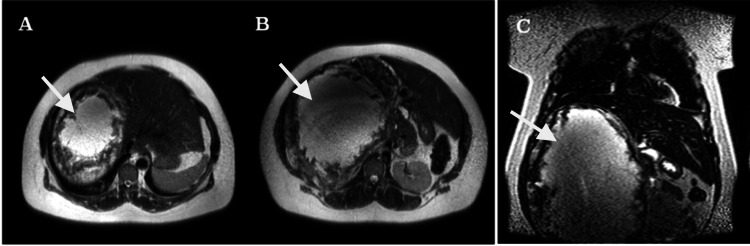
Magnetic resonance imaging axial (A-B) and coronal images (C). The image confirms the presence of a large hyperintense mass (arrows) located in the right hypochondrium.

**Figure 3:**
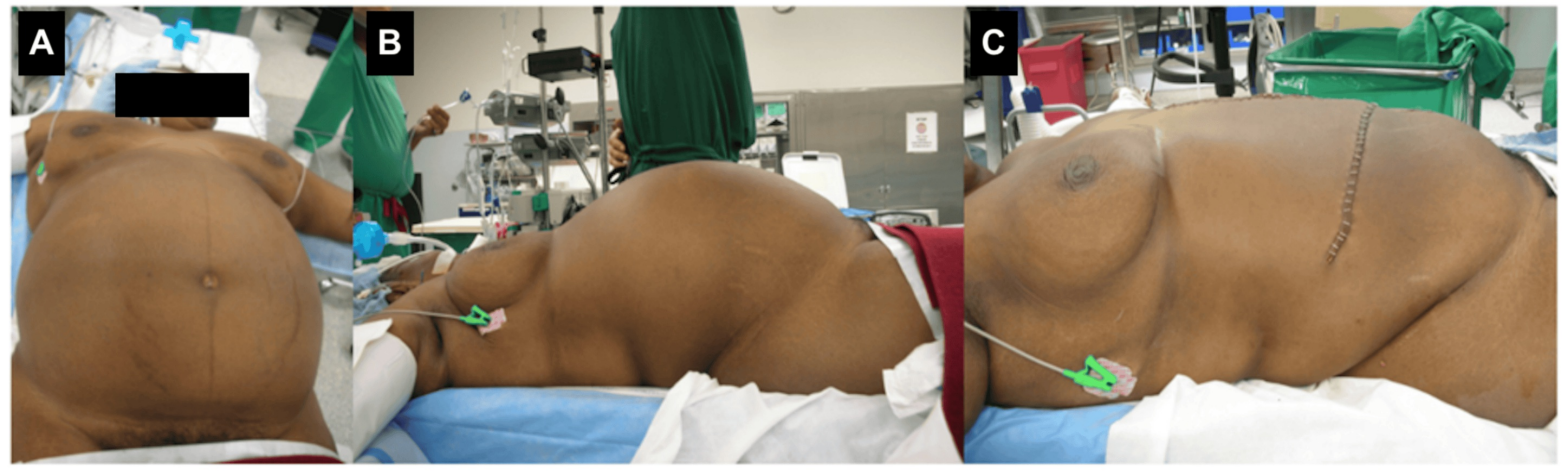
Preoperative and postoperative images. The patient exhibited abdominal enlargement with visible distension prior to surgery (A-B); postoperative image (C) after the resection of the mass.

**Figure 4:**
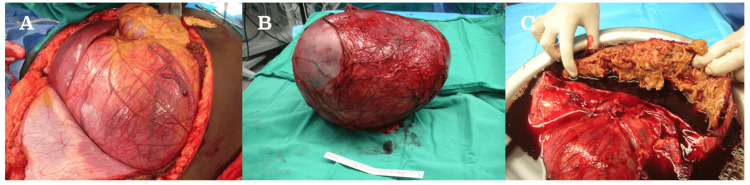
Postsurgical images of the resected mass. Abdominal mass (A) immediately after surgical resection (B-C).

A 47-year-old woman presented to the emergency room with complaints of abdominal fullness. She reported no other associated symptoms, which raised concerns regarding a possible underlying pathology. Her past medical history was otherwise unremarkable, and her past surgical history did not indicate any significant previous interventions. The patient had no known drug allergies and was not taking any medications at the time of her visit. Given the concern for a potential mass, a CT scan of the abdomen was performed (Figure 1), revealing a large mass located on the right side that was displacing the liver. To further characterize the mass, magnetic resonance imaging (MRI) was subsequently conducted (Figure 2), confirming its presence and providing detailed imaging for surgical planning. Based on these findings, a laparotomy was indicated. During the surgical procedure, the mass was resected (Figures 3, 4), and the specimen was sent for histopathological analysis. The results confirmed the diagnosis of adrenal angiosarcoma.

## Discussion

Soft tissue sarcomas comprise 1% of all malignancies. Angiosarcomas, which are rare and aggressive sarcomas with differentiation toward blood or lymphatic endothelium, comprise up to 2% of all these soft tissue sarcomas. Angiosarcomas exhibit a similar distribution between sexes; however, they are more common in elderly White men. These tumors can arise at any anatomic location, with the scalp, breast, and extremities being the most prevalent locations [5,6].

They are mostly spontaneous tumors (cutaneous or visceral angiosarcomas); alternatively, they could also appear as secondary tumors. Radiation-induced angiosarcomas are commonly seen in breast cancer survivors who receive radiotherapy to the chest wall. However, it can occur in any part of the body that has been irradiated previously. Moreover, there have been a few case reports of adrenal angiosarcoma associated with abdominal fibromatosis and exposure to arsenic-containing insecticides and vinyl chloride [7].

There have been limited cases reported for adrenal angiosarcoma, with less than 50 cases reported in the literature, rendering it remarkably rare. Typically, patients present with abdominal pain and/or flank pain, although symptoms can also include weight loss, fatigue, or weakness, or some may remain asymptomatic with incidental findings on imaging, such as an adrenal mass [8]. This variability in presentation underscores the difficulty in diagnosing adrenal angiosarcoma, a highly uncommon and aggressive malignancy.

Above all, cases of adrenal angiosarcomas are exceedingly rare. Ladenheim et al. described a male patient with a history of diabetes mellitus, coronary artery disease, and hypertension whose adrenal tumor measured less than 33 cm [9]. Similarly, Antao et al. presented a case of a male patient with diabetes, left-sided abdominal pain, and fatigue for two months who also had significant anemia [10].

In contrast, we describe a female patient with a rare right adrenal angiosarcoma who presented only with abdominal fullness and denied any type of pain or other previous pathologies. The patient did not have any other risk factors besides obesity. On the abdominal imaging, we observed an exceptionally massive mass related to the disease that stands out compared to the rest of the literature.

Timely diagnosis of adrenal angiosarcoma is crucial due to its aggressive nature and the possibility of confusion with other adrenal neoplasms, lymphomas, melanomas, or metastases from other tumors. Given the rarity of this disease, there are no consensus guidelines for a specific treatment algorithm, further complicating clinical decision-making [8,10].

Adrenalectomy is used for both diagnostic and therapeutic purposes, but early detection remains essential, as the tumor can progress rapidly and aggressively by infiltrating or metastasizing to distant organs. Moreover, patients may experience multiorgan failure as a consequence of the aggressive infiltration, pleural effusion, or, in some cases, severe hemorrhages that can progress to anemia or necrosis associated with cystic formation due to the nature of the tumor. This can necessitate multiple surgical interventions and therapies, thereby significantly worsening the patient's prognosis. For an accurate diagnosis, a CT scan or MRI and biopsy with appropriate immunohistochemical staining are required [3,10,11].

However, due to the level of necrosis and hemorrhage found in the samples, it is sometimes challenging to identify the tumor through biopsy [12]. Additionally, adrenal angiosarcomas have a more epithelioid appearance in histology, unlike most angiosarcomas that present vasoformative patterns. This increases the likelihood of misdiagnosis if a broad immunohistochemical panel is not used [1,10].

The treatment of adrenal angiosarcoma generally involves a multimodal approach due to the aggressive and infiltrative nature of the disease. In most cases, the initial treatment consists of extensive surgical resection, including adrenalectomy and the possible removal of nearby visceral organs affected by tumor infiltration [10]. There is no standardized treatment protocol due to the low incidence of this neoplasm, but patients with resectable masses often undergo surgery, which is associated with improved overall median survival when the disease is localized [8,10]. Conversely, in cases where the tumor had distant metastasis or recurrent adrenal angiosarcoma, the first-line treatment is chemotherapy. Systemic therapy typically involves doxorubicin-based chemotherapy, often combined with ifosfamide or paclitaxel. In metastatic or recurrent cases, targeted therapies such as tyrosine kinase inhibitors have demonstrated some efficacy in soft tissue sarcomas, although evidence specifically for adrenal angiosarcoma remains limited [13,14].

Although surgery and adjuvant chemotherapy have been shown to improve two-year survival in some patients with visceral angiosarcoma, the overall prognosis for adrenal angiosarcoma remains poor, with a five-year survival rate ranging from 24% to 31% [8,15,16]. Poor prognostic factors, such as tumor size greater than 5 cm (as observed in our patient), visceral location, tumor necrosis, and the presence of metastatic disease at diagnosis, contribute to a lower life expectancy [17].

## Conclusions

Adrenal angiosarcoma is an exceptionally rare and aggressive malignancy that presents significant diagnostic challenges due to its nonspecific symptoms and the rarity of the condition. This case highlights the difficulties clinicians face when identifying primary adrenal angiosarcomas, particularly when they present with vague symptoms such as abdominal fullness. Given its aggressive nature, early detection and prompt surgical resection are critical for improving patient outcomes. However, treatment remains complex due to the lack of standardized guidelines, and the overall prognosis is poor, with survival rates being heavily influenced by tumor size, necrosis, and metastasis. Continued awareness and advanced diagnostic approaches, including imaging and biopsy with immunohistochemistry, are essential for accurate diagnosis and better management of this rare malignancy.

## References

[1] Li XM, Yang H, Reng J, Zhou P, Cheng ZZ, Li Z, Xu GH (2017). A case report of primary adrenal angiosarcoma as depicted on magnetic resonance imaging. Medicine (Baltimore).

[2] Weiss DL (1989). Soft tissue tumors. JAMA.

[3] Sung JY, Ahn S, Kim SJ, Park YS, Choi YL (2013). Angiosarcoma arising within a long-standing cystic lesion of the adrenal gland: a case report. J Clin Oncol.

[4] Young WF Jr (2007). The incidentally discovered adrenal mass. N Engl J Med.

[5] Coindre JM, Terrier P, Guillou L (2001). Predictive value of grade for metastasis development in the main histologic types of adult soft tissue sarcomas: a study of 1240 patients from the French Federation of Cancer Centers Sarcoma Group. Cancer.

[6] Rouhani P, Fletcher CD, Devesa SS, Toro JR (2008). Cutaneous soft tissue sarcoma incidence patterns in the U.S.: an analysis of 12,114 cases. Cancer.

[7] Spiker AM, Mangla A, Ramsey ML (2023). Angiosarcoma. StatPearls [Internet].

[8] Fuletra JG, Ristau BT, Milestone B (2017). Angiosarcoma of the adrenal gland treated using a multimodal approach. Urol Case Rep.

[9] Ladenheim A, Tian M, Afify A, Campbell M, Kamangar E (2022). Primary angiosarcoma of the adrenal gland: report of 2 cases and review of the literature. Int J Surg Pathol.

[10] Antao N, Ogawa M, Ahmed Z, Piao J, Poddar N (2019). Adrenal angiosarcoma: a diagnostic dilemma. Cureus.

[11] Grajales-Cruz A, Baco-Viera F, Rive-Mora E (2017). Primary adrenal angiosarcoma: a rare and potentially misdiagnosed tumor. Cancer Control.

[12] Hayashi T, Gucer H, Mete O (2014). A mimic of sarcomatoid adrenal cortical carcinoma: epithelioid angiosarcoma occurring in adrenal cortical adenoma. Endocr Pathol.

[13] McNamara DA, Harmey JH, Walsh TN, Redmond HP, Bouchier-Hayes DJ (1998). Significance of angiogenesis in cancer therapy. Br J Surg.

[14] Wei H, Mao J, Wu Y, Zhou Q (2021). Case report: postoperative recurrence of adrenal epithelioid angiosarcoma achieved complete response by combination chemotherapy with liposomal doxorubicin and paclitaxel. Front Oncol.

[15] Fury MG, Antonescu CR, Van Zee KJ, Brennan MF, Maki RG (2005). A 14-year retrospective review of angiosarcoma: clinical characteristics, prognostic factors, and treatment outcomes with surgery and chemotherapy. Cancer J.

[16] Takizawa K, Kohashi K, Negishi T, Taguchi K, Yamada Y, Nakamura M, Oda Y (2017). A exceptional collision tumor of primary adrenal angiosarcoma and non-functioning adrenocortical adenoma. Pathol Res Pract.

[17] Buehler D, Rice SR, Moody JS (2014). Angiosarcoma outcomes and prognostic factors: a 25-year single institution experience. Am J Clin Oncol.

